# Supplementary material for: Self-supervised pre-training with contrastive and masked autoencoder methods for dealing with small datasets in deep learning for medical imaging
Source: Sci Rep. 2023 Nov 20;13:20260. doi: 10.1038/s41598-023-46433-0 (PMC10662445; doi:10.1038/s41598-023-46433-0)
Supplement: Supplementary file 1 — Supplementary Information. [file 41598_2023_46433_MOESM1_ESM.pdf]

Supplementary Information

Pre-Training Hyperparamter

We use the hyperparameter of the original papers. Only the batch size is adapted due to GPU constraints. We use the maximum possible batch size on our GPU.

Table 1. Hyperparamter MoCo

Parameters	Values
Input Size	512×512
Transforms	crop, horizontal flip, gaussian blur
Number of Crops	2
Size of Crops	224×224
Optimizer	SGD
Batch Size	64
Learning Rate	1e-4
Momentum	0.9

Table 2. Hyperparamter SwAV

Parameters	Values
Input Size	512×512
Transforms	gaussian blur, crop
Number of Crops	2; 6
Size of Crops	224×224; 96×96
Min Scale Crops	0.90; 0.10
Max Scale Crops	1.0; 0.33
Optimizer	Lars
Batch Size	128
Learning Rate	0.15
Weight Decay	1e-6
Sinkhorn Iterations	3
Number Cluster Prototypes	500
Freeze Cluster Prototypes	313

Table 3. Hyperparamter BYOL

Parameters	Values
Input Size	512×512
Transforms	crop, horizontal flip, gaussian blur
Number of Crops	2
Size of Crops	224×224
Optimizer	LARS
Batch Size	64
Learning Rate	1e-3
Weight Decay	1.5e-6

Table 4. Hyperparamter SparK

Parameters	Values
Input Size	512×512
Patch Site	32×32
Mask Ratio	60%
Augmentations	horizontal flip, crop
Batch Size	32
Optimizer	LAMB
Learning rate	Cosine Annealing (peak: 25e-6)

CT Images of the Brain Downstream Task

Table 5. Downstream Task Brain:

Parameters	Values
Format	DICOM
Size	512×512
Slice Thickness	1 mm
Area	Brain
Window Center	35/700 HU
Window Width	80/3020 HU
Tube voltage	100-120 kV
CTDI	33-45
DLP	490-805 mgy·cm
Type	No Contrast-Enhanced
Kernel	Soft Tissue
Scanners	PHILIPS Brilliance iCT 256
	Siemens Somatom Definition AS+
	Siemens Somatom Edge Plus
Gender	Unknown (anonymization)
Age	Unknown (anonymization)
